# Development of the Patient Scale of the Patient and Observer Scar Assessment Scale (POSAS) 3.0: a qualitative study

**DOI:** 10.1007/s11136-022-03244-6

**Published:** 2022-11-10

**Authors:** M. E. Carrière, L. B. Mokkink, Z. Tyack, M. J. Westerman, A. Pijpe, J. Pleat, A. L. van de Kar, J. Brown, H. C. W. de Vet, P. P. M. van Zuijlen

**Affiliations:** 1 Department of Epidemiology and Data Science, Amsterdam UMC (location VUmc), Amsterdam Public Health Research Institute, Amsterdam, The Netherlands; 2 Burn Center and Department of Plastic, Reconstructive and Hand Surgery, Red Cross Hospital, Beverwijk, The Netherlands; 3 Department of Plastic, Reconstructive and Hand Surgery, Amsterdam UMC (location VUmc), Amsterdam Movement Sciences, Amsterdam, The Netherlands; 4 Association of Dutch Burn Centers, Beverwijk, The Netherlands; 5 Child Health Research Centre, University of Queensland, Brisbane, Australia; 6 Burn Center, Royal Brisbane and Women’s Hospital, Brisbane, Australia; 7 Division of Life Science, Amsterdam UMC, VU University, Amsterdam, The Netherlands; 8 Department of Plastic and Reconstructive Surgery, North Bristol NHS Trust, Bristol, UK; 9 Department of Plastic, Reconstructive en Handsurgery, Onze Lieve Vrouwe Gasthuis, Amsterdam, The Netherlands; 10 Pediatric Surgical Centre, Emma Children’s Hospital, Amsterdam UMC, University of Amsterdam, Vrije Universiteit, Amsterdam, The Netherlands

**Keywords:** POSAS, Scar quality, Scar assessment, Content validity, Instrument development, PROM

## Abstract

**Purpose:**

The Patient and Observer Scar Assessment Scale (POSAS) is widely used for measurements of *scar quality*. This encompasses visual, tactile and sensory characteristics of the scar. The Patient Scale of previous POSAS versions was lacking input from patients. Therefore, the aim of this study was to develop the POSAS3.0, Patient Scale with involvement of adults patients with all scar types, complying with the highest clinimetric standards.

**Methods:**

From February 2018 to April 2019, a series of six focus group interviews were performed in the Netherlands and Australia to identify *scar quality* characteristics that adults with scars consider to be important. All focus groups were transcribed, anonymized and analysed using a thematic analysis. Relevant characteristics were formulated into items, resulting in a Dutch and English version of the Patient Scale. These drafts were pilot tested in Australia, the Netherlands and the United Kingdom, and refined accordingly.

**Results:**

A total of 21 relevant *scar quality* characteristics were identified during the focus groups. Two distinct versions of the POSAS3.0, Patient Scale were developed. The *Generic version* contains 16 items and can be used for all scar types, except linear scars. The *Linear Scar version* of the Patient Scale contains the same 16 items, with an extra item referring to the widening of scar margins. All included items are rated on a verbal rating scale with five response options.

**Conclusion:**

Two versions of the POSAS3.0 Patient Scale were developed. Further field tests are being performed to establish the measurement properties and scoring algorithm of the scales.

## Plain English summary

The Patient and Observer Scar Assessment Scale (POSAS) consists of two separate scales: the Patient Scale and the Observer Scale and measures scar quality (i.e. how the scar looks and feels) from both perspectives. Over the years, the POSAS has become a well-known and widely used outcome measurement instrument for measurements of scar quality. However, the Patient Scale of the POSAS was developed by professionals without input from patients. Therefore, we felt a need to improve the Patient Scale by involving a large number of international patients in the development of a new version (i.e. the POSAS3.0). In six consecutive focus groups that took place in the Netherlands and Australia, scar patients discussed what they considered to be the most important characteristics of their scar. Based on these qualitative data, two final versions of the POSAS3.0, Patient Scale were developed and pilot tested. *The Linear Scar version* is used specifically for linear scars, which are scars caused by surgery or trauma, which have a narrow and straight appearance. *The Generic version* can be used for the assessment of all other scar types (except linear scars). Currently, the Patient Scale of the POSAS3.0 is being tested in patients with different types of scars to establish how valid and reliable the scale is able to measure scar quality. More information on the POSAS3.0 scales, and how to obtain and use them can be found on our website (www.posas.org).

## Introduction

*Scar quality* is a construct referring to the visual, tactile and sensory characteristics of the scar [1, 2]. Standardized *scar quality* assessments are necessary to measure the effectiveness of scar treatments, monitor scar maturation over time and identify the need for future treatments in both clinical research and individual patient care. Prior research has demonstrated that professionals and patients might have a different understanding, and therefore operationalization, of the construct *scar quality* [3–6]. As a result, exploration of both perspectives is vital in order to achieve a complete and thorough scar evaluation. For this reason, the POSAS was the first to include both perspectives using two separate scales: the Patient Scale and the Observer Scale [7, 8]. Several systematic reviews on *scar quality* scales have rated the POSAS as the best available scale, because it has good measurement properties, and includes a separate assessment for observers (often professionals), as well as for patients [9–12]. However, the POSAS2.0, which dates back to 2005, has several limitations. First and foremost, the Patient Scale was developed by professionals without input from patients living with scars. Second, the Observer Scale was developed in the Netherlands and could be criticised for not having a more global perspective. As a result, we determined a need to improve the content validity (i.e. comprehensiveness of content, as well as relevance and comprehensibility of items), and the generalizability of the POSAS2.0. This was done by including a large number of international experts: patients as well as professionals in the development of a new version, i.e. the POSAS3.0. The objective of the present study was to develop the Patient Scale of the POSAS3.0 for measurements of *scar quality* in adults with all types of scars. The POSAS3.0, Observer Scale has been developed using an extensive international Delphi study, which will be published separately.

## Methods

**Fig. 1:**
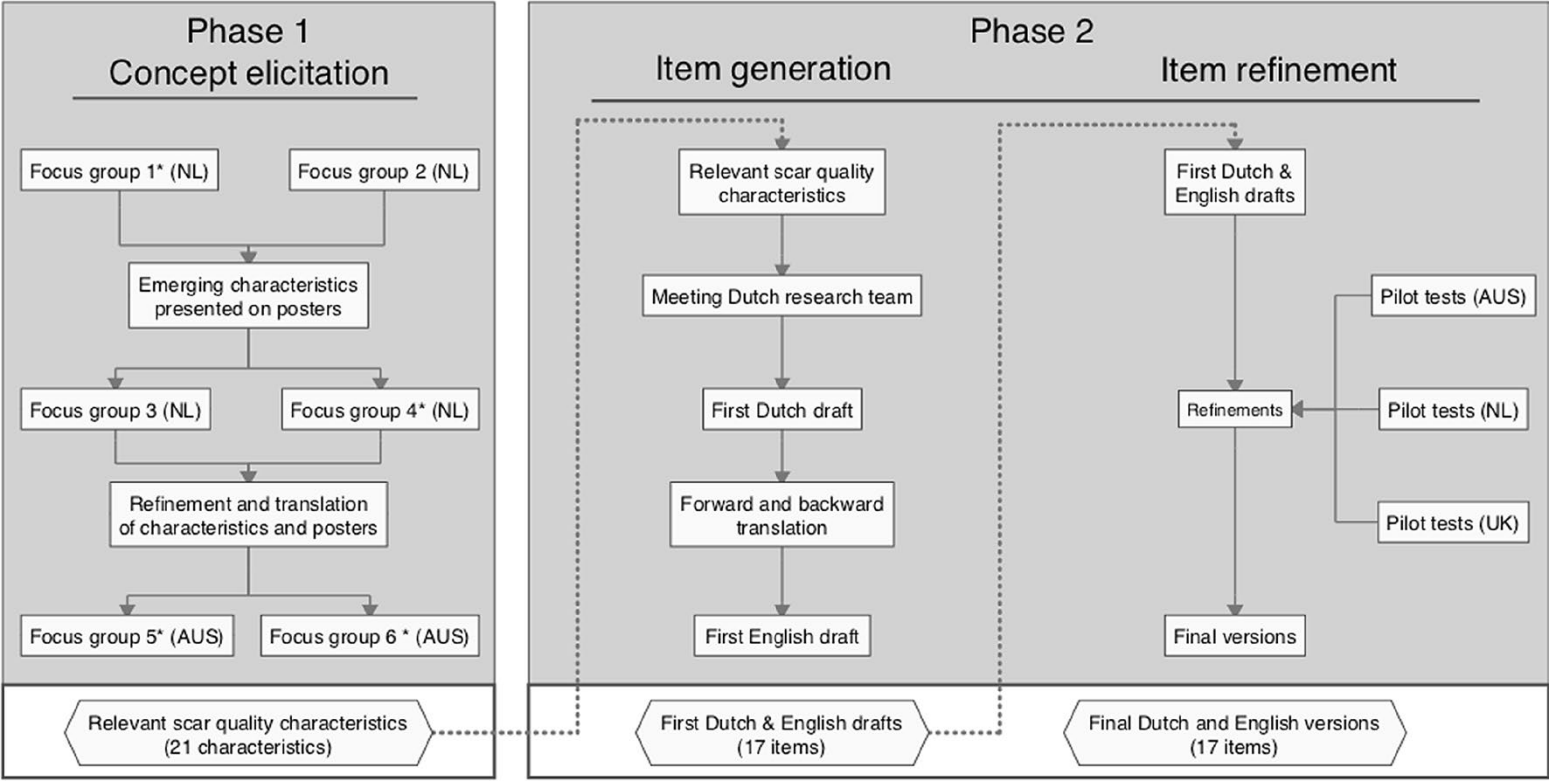
Flowchart illustrating the study procedure. *Focus groups in which individuals with scars caused by burns and necrotizing fasciitis took part. The remaining focus groups included individuals with surgical (linear), traumatic, and acne scars

This study consisted of two phases: (1) concept elicitation, and (2) item generation and refinement. Phase 1 took place in the Netherlands and Australia, and phase 2 took place in Australia, the Netherlands, and the United Kingdom. Figure 1 shows the flowchart of the study procedure. This study was guided by a steering committee consisting of an international team of clinimetric experts, clinicians and qualitative researchers. All authors of this publication were included in the steering committee. The guidelines for content validity studies provided by COSMIN and ISPOR PRO good Research practice were followed [13, 14]. Ethical approval was obtained from the Amsterdam UMC (location VUmc) Research Ethics Committee (2017/552) and from the Royal Brisbane and Women’s Hospital’s Human Research Ethics Committee (45062).

### Definition of the construct: *scar quality*

Before the start of the focus groups, the international POSAS3.0 steering committee reached consensus on the definition of the construct of the POSAS. The POSAS aims to measure *scar quality*. *Scar quality* is defined as the visual, tactile and sensory characteristics of a scar [1, 2]. A scar was defined as ‘a mark remaining on the skin after injured tissue has healed’. *Scar quality* captures the first two health domains included in the conceptual model of Wilson and Cleary: the biological and physiological variables (i.e. visual and tactile characteristics), and symptom status (i.e. sensory characteristics) [15]. In terms of symptom status, we consider only the symptoms that are related to and experienced within the scar (e.g. itch) to be part of the construct *scar quality*. Symptoms caused by the trauma or injury that can be experienced in the body as a whole are referred to as *systemic symptoms* (i.e. fatigue), and are not part of this construct. This has been decided because the POSAS aims to be comprehensive, yet concise, and, therefore, easily applicable in clinical practice. Although *systemic symptoms*, *functional status* and *overall quality of life* are considered very important health domains within scar evaluation, these constructs were considered beyond the scope of the POSAS (i.e. *scar quality*) and should be measured using alternative outcome measurement instruments.

### Phase 1: concept elicitation

#### Design

From February 2018 to April 2019, a series of six focus group interviews were performed to explore the characteristics of *scar quality* that individuals with scars consider to be important. The focus groups took place in conference rooms in three different hospitals: the Red Cross Hospital, Beverwijk, the Netherlands (first, second and fourth group); the Amsterdam UMC (location VUmc), the Netherlands (third group); and the Royal Brisbane and Women’s Hospital, Australia (fifth and sixth group). For the preparation of focus groups, topic guides were used. The topic guide for the first focus groups was based upon the results of a systematic review on outcome measurement instruments of *scar quality* [2]. The topic guides for the following focus groups were refined on the basis of the results and experiences of previous focus groups. Each focus group was moderated by two authors (either MW or ZT). The lead author (MC) was present in all focus groups. Both MW and ZT have many years of experience in conducting qualitative research methods. MC has completed a certified course in qualitative research for the purpose of this study. In the Dutch focus groups, an additional member of the research team (LM) with clinimetric expertise was present to take notes on group dynamics and non-verbal communication between participants. None of the researchers present during the focus groups had a relationship with participants prior to study commencement. All focus groups were audio recorded.

#### Participants

Participants were English- and Dutch-speaking adults (≥ 18 years), living with scars resulting from burns, surgery, infection, trauma and acne. Participant selection was based on purposive sampling to ensure that participants represented a wide range of personal characteristics (e.g. gender, age, skin type) and scar characteristics (e.g. scar size, location and age of scar). Given a target of 8–10 participants per focus group, we recruited up to 12 patients per group to allow for non-attendance. Each participant took part in one focus group. Participants were recruited from scar, trauma, or plastic surgery outpatient clinics at two locations in the Netherlands (the Red Cross Hospital in Beverwijk and the Amsterdam UMC) and one location in Australia (the Royal Brisbane and Women's Hospital). The Dutch burn patient support group also served as a source of recruitment. Patients received a flyer with study information and were asked to give their written consent for the research team to contact them by telephone. During this call, potential participants were screened to confirm eligibility. Patients with scars caused by burns or necrotizing soft tissue infection (NSTI) were placed in the same focus groups due to their similarities in wound treatment and resulting scar patterns. Patients with surgical scars were placed in separate focus groups from those with burns or NSTI. Patients with keloids were divided into either surgical or burn focus groups, based on the cause of the keloid. All patients were informed about the confidential nature of the study and the possibility to withdraw from it at any time. All patients provided written informed consent for participation and received travel reimbursements.

#### Content of the focus group interviews

All focus groups lasted 2 h. At the beginning, MC introduced the research team, presented the study aims and agenda and initiated an introduction round so that participants could get to know one another. Throughout all meetings, food and beverages were available, and halfway, participants were offered a short break of approximately 10 min. During the first and second focus groups, we asked participants the general question: “What are the things that come to mind when you think about the look and the feel of your scar?”, thereby specifically focussing on the sensation and physical feeling of the scar rather than on the psychological emotions associated with it, or the impact on quality of life. Participants were asked to write all the characteristics on sticky notes, which in turn were discussed and categorized on a whiteboard. Following an iterative approach, the characteristics that emerged in the first two focus group were presented on posters in the third and fourth focus groups. Participants were asked to highlight the characteristics on the posters that were relevant to them using stickers and to provide synonyms for these characteristics. Additionally, they were asked to add new notes with additional characteristics that they perceived as lacking. Next, individuals were invited to collectively elaborate on their experiences. The same method was used for the fifth focus group in Australia using refined and translated posters. In the sixth, and final, focus group, participants were asked to comment on a summary of the identified *scar quality* characteristics by explaining what they thought the characteristics meant in general and what the characteristics meant to themselves in the context of their own experiences. Finally, participants were asked to prioritise the five most important characteristics. This provided insight into the order of importance of the identified characteristics according to our study sample.

#### Analysis

All recorded focus group discussions were transcribed ad verbatim and anonymized by MC. Member checking was not performed due to confidentiality issues. The transcripts of the first two focus groups were coded independently by MC and LM. All of the subsequent transcripts were coded by MC and checked by either LM or ZT. A thematic analysis was used, following the steps described by Braun and Clark (i.e. familiarisation with the data; generating codes; searching for themes; reviewing, defining, and naming themes; and producing the report) [16]. All characteristics of *scar quality*, including the different ways to describe them, were extracted from the transcripts. Subsequently, the number of characteristics was reduced by removing characteristics that were not considered relevant for the construct *scar quality*. No software was used for the analysis.

### Phase 2: item generation and refinement

#### Item generation

To optimize implementation in clinical practice, it was important to keep the POSAS user-friendly, and as concise as possible by only including key attributes of scar quality that can be assessed by looking at and feeling the scar. During a meeting of the Dutch research team, the relevant characteristics that were identified in phase one were formulated into items with answering options using the wording that participants used. Two patient-researchers, one with burn scars and one with scars caused by NSTI, who were also present in one of the focus groups joined this meeting to ensure that the patient perspective was well understood and presented in the scale. This led to the Dutch draft of the Patient Scale. The similarity of the content of the Dutch and English scales was maintained through the use of a forward and backward translation on the Dutch Patient Scale, as described by Beaton [17].

#### Item refinement

The resulting drafts were pilot tested at the outpatient clinics of (1) the Royal Brisbane and Women’s Hospital in Brisbane, Australia; (2) the Red Cross Hospital in Beverwijk, the Netherlands and (3) Southmead Hospital in Bristol, United Kingdom. Pilot tests were performed by either MC, and/or ZT, or JP. The Three-Step Test-Interview (TSTI) method was used: participants were asked to verbalize their thoughts while filling in the scale, to clarify any non-verbal reactions they may have had, and to explain their responses and address any additional issues related to filling in the questionnaire. In this way, information was gained on a person’s understanding, interpretation and scoring of the included items [18]. Revisions deemed necessary based upon pilot test data were applied in both languages to maintain consistency between versions.

## Results

The results of this qualitative study are reported in two articles. The current article describes the development process. An additional, complementary, article provides an elaboration on and clarification for choices made during the development regarding item selection, formulation and merging [19].

### Phase one: concept elicitation

#### Participants

A total of 55 patients agreed to participate prior to the focus groups. Of those patients, a total of 43 participants actually attended one of the meetings. Table 1 shows the characteristics of participants and their scars.

**Table 1:** Characteristics of participants and their scars included in the focus groups and pilot tests

	Phase 1						Phase 2		
	FG 1 (NL)	FG 2 (NL)	FG 3 (NL)	FG 4 (NL)	FG 5 (AUS)	FG 6 (AUS)	Pilot tests (AUS)	Pilot tests (NL)	Pilot tests (UK)
Participants, *n*	7	6	6	9	8	7	5	5	5
Male	3	2	1	2	6	5	4	3	1
Female	4	4	5	7	2	2	1	2	4
Age in years, *median (range)*	50 (37–60)	48 (25–79)	38 (24–81)	38 (20–61)	43 (19–70)	44 (21–72)	25 (18–49)	53 (38–72)	34 (23–47)
Cause of scars*, *n*									
Burns	6			8	8	7	5	1	1
Surgery		4**	3					3	1
NSTI	2			1				1	
Acne		1**	1**						
Trauma		1	2						2
Other (piercing)									1**
Age of scars in years, *median (range)*	3.0 (1.0–37.0)	5.0 (0.5–11.0)	14.0 (0.5–18.0)	15.0 (0.5–35.0)	1.2 (0.1–3.0)	1.0 (0.1–3.0)	0.2 (0.1–1.0)	34.0 (0.5–56.0)	1.2 (0.3–6.0)
TBSA in %, *median (range)*	19 (10–67)	N/A	N/A	18 (2–68)	17 (1–60)	15 (1–33)	2 (1–16)	40	20
Location of scars, *n*									
Trunk		5	3	3				2	
Arm(s)	1	1		1			1		
Hand(s)							1		1
Leg(s)	1		3		3	1		2	1
Feet					1		1	1	
Face									2
≥ 2 locations***	5			5	4	6	2		1

*FG* focus group, *NSTI* necrotizing soft tissue infection, *TBSA* total burned surface area, *SD* standard deviation, *N/A* not applicable

*By “cause of scars”, the initial cause of the wound, which eventually resulted in a scar, is meant. If the initial wound was caused by burns and received surgery in a later stage, the cause of the scar is noted here as being burns

**In phase 1, three participants had keloids, of which two were caused by acne, and one was caused by surgery. In the UK pilot tests, one participant had a keloid, which was caused by a piercing

***Nearly all of these 20 scars were extensive burn scars, of which six also covered the face

#### Content of the Patient Scale

**Fig. 2:**
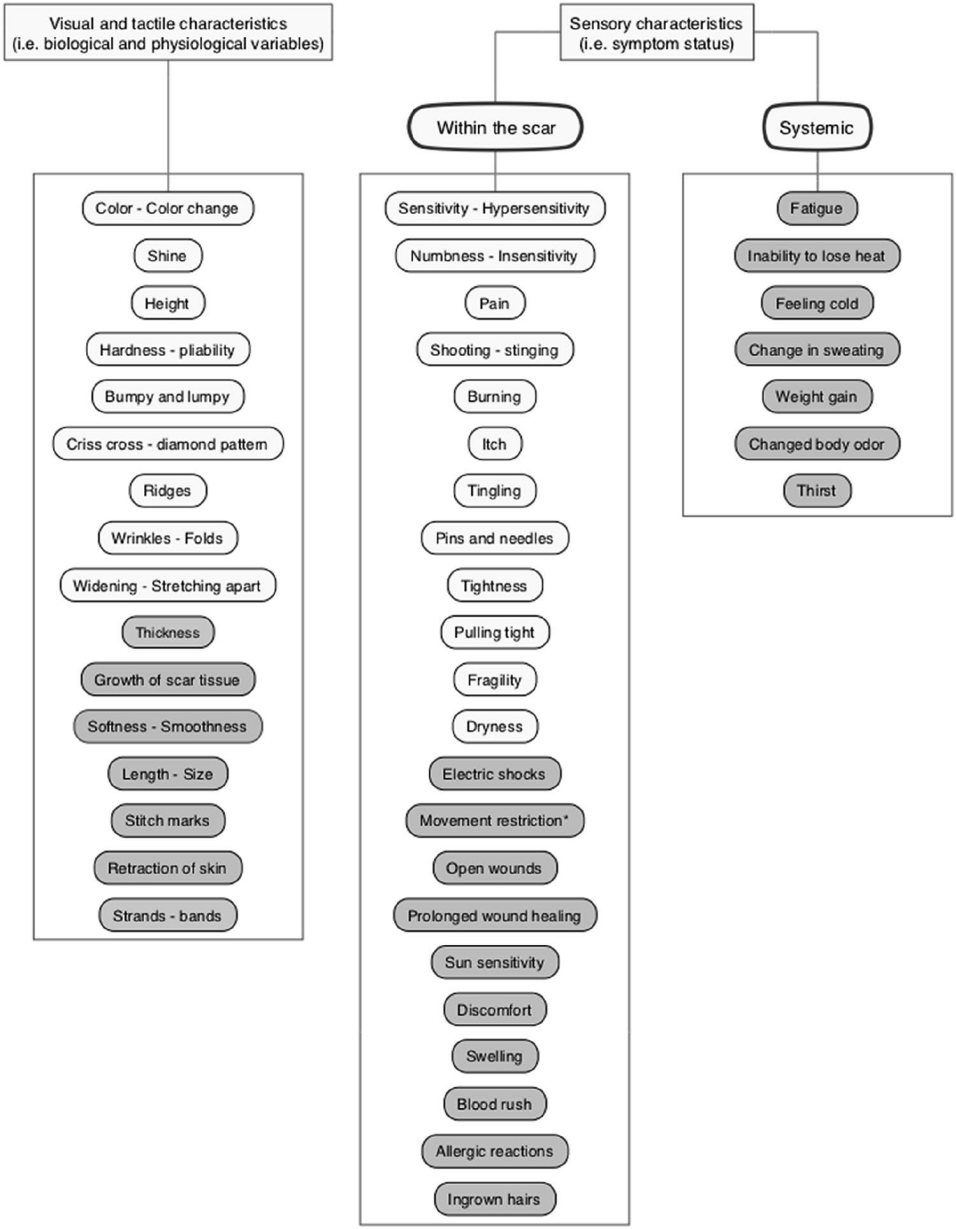
Characteristics that were identified during phase 1 of the study (i.e. concept elicitation), categorized into two groups: (1) visual and tactile characteristics and (2) sensory characteristics. The sensory group is further subdivided into symptoms that occur within the scar, and symptoms that are caused by the trauma or injury and can be experienced in the body as a whole (i.e. *systemic symptoms*). Characteristics that are marked grey were not included in the final version(s) of the POSAS3.0, Patient Scale

A total of 45 different characteristics were identified in phase 1, as shown in Fig. 2. Based on the different domains of health outcomes, as defined by Wilson and Cleary [15], these characteristics were categorized into two groups: (1) Visual and tactile characteristics (i.e. biological and physiological variables), and (2) sensory characteristics (i.e. symptom status). The symptom group was further subdivided into symptoms that are related to and experienced within the scar area (i.e. indicators of *scar quality*), and symptoms caused by the trauma or injury that can be experienced in the body as a whole (i.e. *systemic symptoms*). *Systemic symptoms* (e.g. fatigue) were excluded because they were not considered part of the construct *scar quality*. *Scar quality* characteristics that were not considered to be relevant enough by focus group participants, or were infrequently mentioned (e.g. ingrown hairs) were also excluded. This resulted in a final set of 21 relevant *scar quality* characteristics. All 21 relevant characteristics had been identified by the completion of the second focus group. Therefore, data saturation was achieved after the second focus group. A detailed description of the rationale for exclusion and inclusion of characteristics and item wording can be found in a separate publication [19].

### Phase 2: item generation and refinement

#### Item generation

All items included in the POSAS3.0 contribute to the construct *scar quality*, and therefore, the POSAS3.0 is based on a formative model. During the Dutch research meeting, the initial 21 relevant characteristics of *scar quality* were formulated into 17 items. At this time, it also became clear that the development of two distinct POSAS3.0 Patient Scales was required: the *Generic* version for use in all non-linear scars (e.g. those caused by burns, infection and trauma), and the *Linear Scar version* specific for use in linear scars caused by surgery or trauma. The necessity for two separate scales arose as one particular item, the widening of scar margins, was unique for patients with linear scars. Patients with non-linear scars did not understand or recognize the meaning of this item with respect to their own scars.

#### Item refinement

Table 1 shows the characteristics of the participants who took part in the pilot tests. During the pilot tests, the most important points of discussion revolved around combining or separating similar items, and the wording of items [19].

#### Response options

**Fig. 3:**
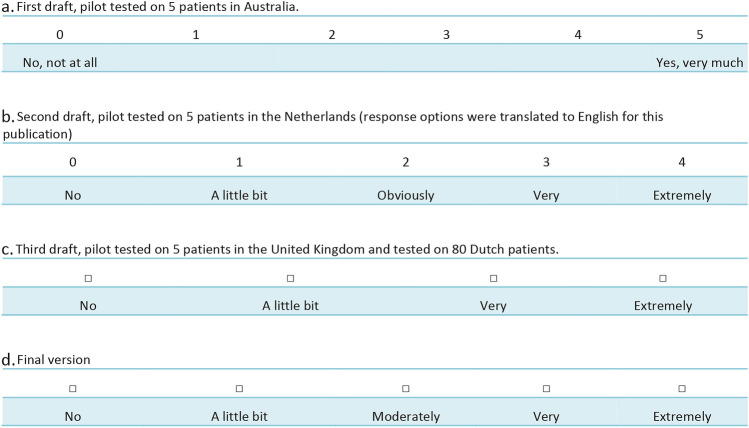
Development of the response options throughout phase 2 of this study

Figure 3 shows the different stages of the development of the response options. The first draft included a 6-point numeric scale. Based on the preferences of pilot test participants, the response options were changed to a verbal scale (i.e. with words attached to each category instead of numbers), as shown in Fig. 3b. Participants favoured the use of words because they provide clarity about the meaning of each category. Next, the response options were reduced to a 4-point verbal scale, because some participants considered it challenging to discern between the categories “Obviously” and “Very” (Fig. 3c). At the time, this set of response options was deemed final and was used for the first field tests in the Netherlands. However, many participants reported that they missed a response option between “a little” and “very”. Therefore, after being used on 80 Dutch participants, the ultimate decision was made to change the options back to the 5-point verbal scale, but changing the word “Obviously” to “Moderately”, resulting in the response options shown in Fig. 3d.^1^

#### Final versions of the Patient Scale

The final versions of the POSAS3.0, Patient Scale can be downloaded on the POSAS website (www.posas.nl). The Generic version contains 16 items. The Linear Scar version contains the same 16 items, with one extra item referring to the widening of scar edges (i.e. 17 items in total). All included items are rated on a verbal rating scale with five response options. The scale design was done by a professional company specialized in science communication.

## Discussion

This study described the process of development and refinement of the Patient Scale of the POSAS3.0, which is suitable for measurements of *scar quality* in adults with all types of scars. In a second, complementary, article, we clarify and elaborate on the choices that were made during the development of the scale regarding the selection, formulation and merging of items, using an in-depth insight into our qualitative data [19]. The construct *scar quality* encompasses visual, tactile and sensory characteristics of the scar, and does not include *systemic symptoms* or the impact that scars may have on the quality of life of individuals living with them [19].

The main reason for initiating this study was the lack of patient input in the development of the previous POSAS versions. Using a qualitative approach, this study aimed to establish how individuals with scars value and define the characteristics of *scar quality*. By including *scar quality* characteristics that individuals with scars find most important, using the language that they used to describe them with, we aimed to improve the content validity of the POSAS. For this reason, it is our intention to have the old versions of the POSAS replaced with the POSAS3.0 by users. The rigorous methodological approach used in the development of POSAS3.0 led to considerable differences in scale content compared to earlier versions. First, the total number of items included in the POSAS3.0, Patient Scale is higher (16 or 17 items) than in previous versions (6 items). The greatest difference is attributed to an increase in the number of sensory items—components which only patients are able to perceive and rate. The POSAS3.0, Patient Scale includes 11 sensory items, whereas the previous version only includes two sensory items (i.e. pain and itch). This difference highlights the added value in asking the patients for input. Secondly, the 10-point rating scale has been changed to a verbal rating scale with 5 response options. This decision was made because patients often found it difficult to discriminate between 10 different response options. This was also supported by Rasch analyses on the POSAS2.0, Patient Scale, which demonstrated a malfunctioning of the 10-point scale and suggested a reduction of the number of answering options [20, 21]. Third, the POSAS3.0 contains clear instructions and questions in lay language, facilitating independent scale completion, whereas in previous versions, health care professionals often needed to provide additional clarification of questions as the questionnaire was completed by the patient. Despite the marked differences between POSAS3.0 and earlier versions, there are also similarities in scale content. Four items included in the POSAS2.0 (i.e. pain, itch, colour and irregularity) are also integral to the new version. Two remaining items, stiffness and thickness, are presented slightly different in the new scale, because patients preferred the use of the terms ‘hardness’ and ‘height’, respectively.

This study is the first international qualitative investigation into the patient’s perspective on *scar quality*. A fundamental strength of this study is its thorough and iterative methodological approach. The research team was composed of clinimetric experts, clinicians and qualitative researchers, but patients with scars had input in all phases of the development process. In this way, we incorporated the theoretical knowledge regarding *scar quality*, scale development and qualitative research with the diverse patient experience. In preparation for this study, an extensive systematic review of the literature was conducted [2], which provided a complete overview of all the characteristics of *scar quality* that had ever been measured. All focus groups were guided by experienced and certified moderators and surgical patients were recruited from a range of different surgical specialties (i.e. gynaecology, trauma, plastic surgery), which contributed to the generalizability of our results.

A potential disadvantage of focus groups in comparison to individual interviews is that participants are more likely to adopt and affirm each other’s opinions. We cannot exclude that this occurred in our focus groups. However, advantages of the focus group process are the ability for participants to identify and clarify their opinion, as well as to compare and revise it based upon the opinions of others [22]. This may have led to more ideas about—and deeper insight into—the construct *scar quality* than would have been identified in one-on-one interviews [23–25]. Two distinct versions of the POSAS3.0 Patient scale were developed for different scar types (i.e. the Generic version and the Linear Scar version) to avoid misinterpretation of the item *scar widening* by patients with non-linear scars. A limitation of our study is that a third POSAS version was deemed necessary for keloid patients, but we did not include enough keloid participants in the first phase of our study (*n* = 3) to adequately develop this version. Further research is necessary to address this. Another limitation related to our study population is that the majority had burn scars. However, as burn scars are considered as one of the most complex and burdensome scars, we believe that no relevant characteristics have been missed. Currently, we are conducting follow-up studies with the Patient Scale of the POSAS3.0. In order to facilitate the calculation of the total score for *scar quality* (e.g. a weighted scoring algorithm), we aim to determine the importance of individual items for the construct *scar quality*. In addition, another follow-up study is being performed to establish the measurement properties (i.e. reliability, measurement error, responsiveness [26]) of the POSAS3.0 Patient Scale. These findings will indicate if the qualitative methodology used in this development study have indeed led to improved measurement properties of the POSAS3.0 in comparison to the previous POSAS version. Furthermore, an international Delphi study has been conducted to better understand and evaluate the professional perspective on *scar quality*. This led to the development of the Observer Scale of the POSAS3.0. Together, both scales fo the POSAS3.0 will provide a comprehensive *scar quality* assessment in adults with all scar types. More information on how to obtain and use the POSAS3.0 scales can be found on our website (www.posas.org).

## Conclusion

Using qualitative methods, two versions of the POSAS3.0, Patient Scale—the *Generic* version, and the *Linear scar version*—were developed. Currently, further field tests are being performed in order to establish the measurement properties and scoring algorithm of the scales.

## Footnotes

1 The 80 field tests that were collected were disregarded and the field tests study was restarted with the final set of response options.
